# Non‐HIV‐infected patients with *Pneumocystis* pneumonia in the intensive care unit: A bicentric, retrospective study focused on predictive factors of in‐hospital mortality

**DOI:** 10.1111/crj.13463

**Published:** 2022-01-10

**Authors:** Yuqiong Wang, Xu Huang, Ting Sun, Guohui Fan, Qingyuan Zhan, Li Weng

**Affiliations:** ^1^ China‐Japan Friendship School of Clinical Medicine Peking University Beijing China; ^2^ Department of Pulmonary and Critical Care Medicine, Center of Respiratory Medicine China‐Japan Friendship Hospital Beijing China; ^3^ China‐Japan Friendship School of Clinical Medicine Capital Medical University Beijing China; ^4^ Institute of Clinical Medical Sciences China‐Japan Friendship Hospital Beijing China; ^5^ Institute of Respiratory Medicine Chinese Academy of Medical Sciences, National Clinical Research Center for Respiratory Disease, National Center for Respiratory Disease Beijing China; ^6^ Medical Intensive Care Unit, State Key Laboratory of Complex Severe and Rare Diseases Peking Union Medical College Hospital, Peking Union Medical College, Chinese Academy of Medical Sciences Beijing China

**Keywords:** HIV‐negative (non‐HIV), mortality, *Pneumocystis* pneumonia, prognostic factor

## Abstract

**Background:**

The incidence of *Pneumocystis* pneumonia (PCP) among patients without human immunodeficiency virus (HIV) infection continues to increase. Here, we identified potential risk factors for in‐hospital mortality among HIV‐negative patients with PCP admitted to the intensive care unit (ICU).

**Methods:**

We retrospectively analyzed medical records of 154 non‐HIV‐infected PCP patients admitted to the ICU at Peking Union Medical College Hospital (PUMCH) and China‐Japan Friendship Hospital (CJFH) from October 2012 to July 2020. Clinical characteristics were examined, and factors related to in‐hospital mortality were analyzed.

**Results:**

A total of 154 patients were enrolled in our study. Overall, the in‐hospital mortality rate was 65.6%. The univariate analysis indicated that nonsurvivors were older (58 vs. 52 years, *P* = 0.021), were more likely to use high‐dose steroids (≥1 mg/kg/day prednisone equivalent, 39.62% vs. 55.34%, *P* = 0.047), receive caspofungin during hospitalization (44.6% vs. 28.3%, *P* = 0.049), require invasive ventilation (83.2% vs. 47.2%, *P* < 0.001), develop shock during hospitalization (61.4% vs. 20.8%, *P* < 0.001), and develop pneumomediastinum (21.8% vs. 47.2%, *P* = 0.001) and had higher Acute Physiology and Chronic Health Evaluation (APACHE) II scores on ICU admission (20.32 vs. 17.39, *P* = 0.003), lower lymphocyte counts (430 vs. 570 cells/μl, *P* = 0.014), and lower PaO2/FiO2 values (mmHg) on admission (108 vs. 147, *P* = 0.001). Multivariate analysis showed that age (odds ratio [OR] 1.03; 95% confidence interval [CI] 1.00–1.06; *P* = 0.024), use of high‐dose steroids (≥1 mg/kg/day prednisone equivalent) during hospitalization (OR 2.29; 95% CI 1.07–4.90; *P* = 0.034), and a low oxygenation index on admission (OR 0.99; 95% CI 0.99–1.00; *P* = 0.014) were associated with in‐hospital mortality.

**Conclusions:**

The mortality rate of non‐HIV‐infected patients with PCP was high, and predictive factors of a poor prognosis were advanced age, use of high‐dose steroids (≥1 mg/kg/day prednisone equivalent) during hospitalization, and a low oxygenation index on admission. The use of caspofungin during hospitalization might have no contribution to the prognosis of non‐HIV‐infected patients with PCP in the ICU.

## 1 INTRODUCTION

*Pneumocystis* pneumonia (PCP) is a potentially life‐threatening fungal infection in immunocompromised patients caused by *Pneumocystis jiroveci* (*P. jiroveci* [Pj]).^1^ With advances in highly active antiretroviral therapy and routine prophylaxis, the incidence of PCP among patients infected with human immunodeficiency virus (HIV) has markedly decreased worldwide.^2^, ^3^ Nevertheless, PCP continues to garner attention because of the significant increases in its incidence in patients who are receiving corticosteroids, biological agents, or immunosuppressants.^4^, ^5^ PCP has become more common among this population as a result of treatment changes, such as the increased use of immunosuppressive agents to treat patients with malignancies, inflammatory diseases, and solid organ transplants (SOTs). HIV‐negative patients with PCP typically present with rapidly progressive respiratory failure,^6^, ^7^, ^8^ and the mortality rate ranges from 33.3% to 69.3%.^8^, ^9^, ^10^, ^11^, ^12^, ^13^, ^14^ These patients require intensive care more often.^15^ To date, few studies have been performed to identify predictive factors of PCP in non‐HIV‐infected patients. Therefore, the main purpose of this study was to share our experience with PCP treatment at two tertiary referral centers and to identify potential risk factors for in‐hospital mortality among HIV‐negative patients with PCP. We performed a retrospective, bicentric, observational study of consecutive patients with confirmed PCP who were admitted to the intensive care unit (ICU) from October 2012 to July 2020.

## 2 METHODS

### 2.1 Study design and patients

This bicentric, retrospective study included ICU inpatients at two academic medical institutions from October 2012 to July 2020. The institutions were Peking Union Medical College Hospital (PUMCH, a tertiary care affiliated teaching hospital with more than 2000 beds) and China‐Japan Friendship Hospital (CJFH, a tertiary care center with approximately 1600 beds). Data were retrospectively gathered from medical records at these two hospitals by the investigators.

Our inclusion criteria were as follows: (1) PCP diagnosed from sputum, trachea aspirate, or bronchoalveolar lavage fluid (BALF) samples by silver methylamine staining or polymerase chain reaction (PCR); (2) negative serum HIV tests; (3) a potential relationship between immune deficiency and the development of PCP; and (4) ICU admission due to respiratory insufficiency.

Patients with incomplete medical information, with Pj colonization, who were less than 18 years of age or who were pregnant were excluded. As for the variable selection of regression model, Acute Physiology and Chronic Health Evaluation (APACHE) II score includes age and multiple physiological indicators, which overlapped with other variables we included, so it was not included in the regression analysis. Fever, as a clinical symptom, was not specific, so it was not included in the regression model. Mechanical ventilation and shock were considered indicators of patients' outcome and were not included in the regression analysis. To maintain patient anonymity and confidentiality, we did not collect identifying information, and only the researchers involved in this study evaluated the data. The need to obtain written informed consent was waived by the ethics committee due to the retrospective nature of the analysis.

### 2.2 Definitions

1. Patients who met the following criteria were defined as having PCP: (1) the presence of relevant pulmonary symptoms (dry cough, fever, or progressive dyspnea); (2) radiological findings (pulmonary infiltration) consistent with PCP^16^; (3) detection of Pj in respiratory secretions, such as sputum, tracheal aspirate, and BALF, or in biopsy tissue by silver methylamine staining or PCR.^17^, ^18^, ^19^ PCP diagnostic tests were performed when there was a strong suspicion of infection.
2. Pj colonization was defined as the positive detection of Pj DNA by PCR or silver methoxamine staining in the absence of relevant signs or radiographic evidence.^20^
3. Coinfection was defined as the identification of other pathogens in respiratory secretions positive for Pj.
4. Immunocompromised patients were defined as patients with malignant tumors (hematological malignancies or solid tumors), those undergoing bone marrow or solid organ transplantation, and those receiving immunosuppressive treatment for autoimmune or other diseases.

### 2.3 Data collection and analysis

We reviewed the clinical data of patients with PCP. Demographic, clinical, and laboratory data, including age, sex, underlying medical conditions, immune status, initial symptoms, microbiological findings, the duration from the onset of symptoms to diagnosis, antibiotics used, mechanical ventilation parameters, complications, and disease outcome, were collected. Mechanical ventilation data included the type of oxygen therapy or ventilation support used at admission, respiratory support mode, partial pressure of arterial oxygen/inspiratory fraction of oxygen (PaO2/FiO2) at admission, intubation duration, platform pressure, and tidal volume at admission. The primary outcome of our study was in‐hospital mortality. All clinical data were anonymized at the time of collection by two independent investigators to ensure patient privacy. The two investigators received simultaneous training. In cases of disagreements between the investigators, a consensus was reached through discussion or adjudication by a third party.

Continuous variables are expressed as the mean ± SD and median (interquartile range [IQR]). Categorical variables are expressed as numbers and percentages. Student's *t* test and the Mann–Whitney *U* test were used to compare quantitative variables. The chi‐square test and Fisher's exact test were used to compare categorical variables.

We performed a univariate analysis of in‐hospital mortality for non‐HIV‐infected patients with PCP. Statistical significance was defined as a *P* value < 0.05. Covariates with *P* < 0.05 and clinically important variables were considered for inclusion in the multivariate logistic regression analysis to examine correlations between in‐hospital mortality and each variable. Four explanatory variables were included in the multivariate logistic regression. Statistical analysis was performed with IBM SPSS Statistics version 25.

**FIGURE 1:**
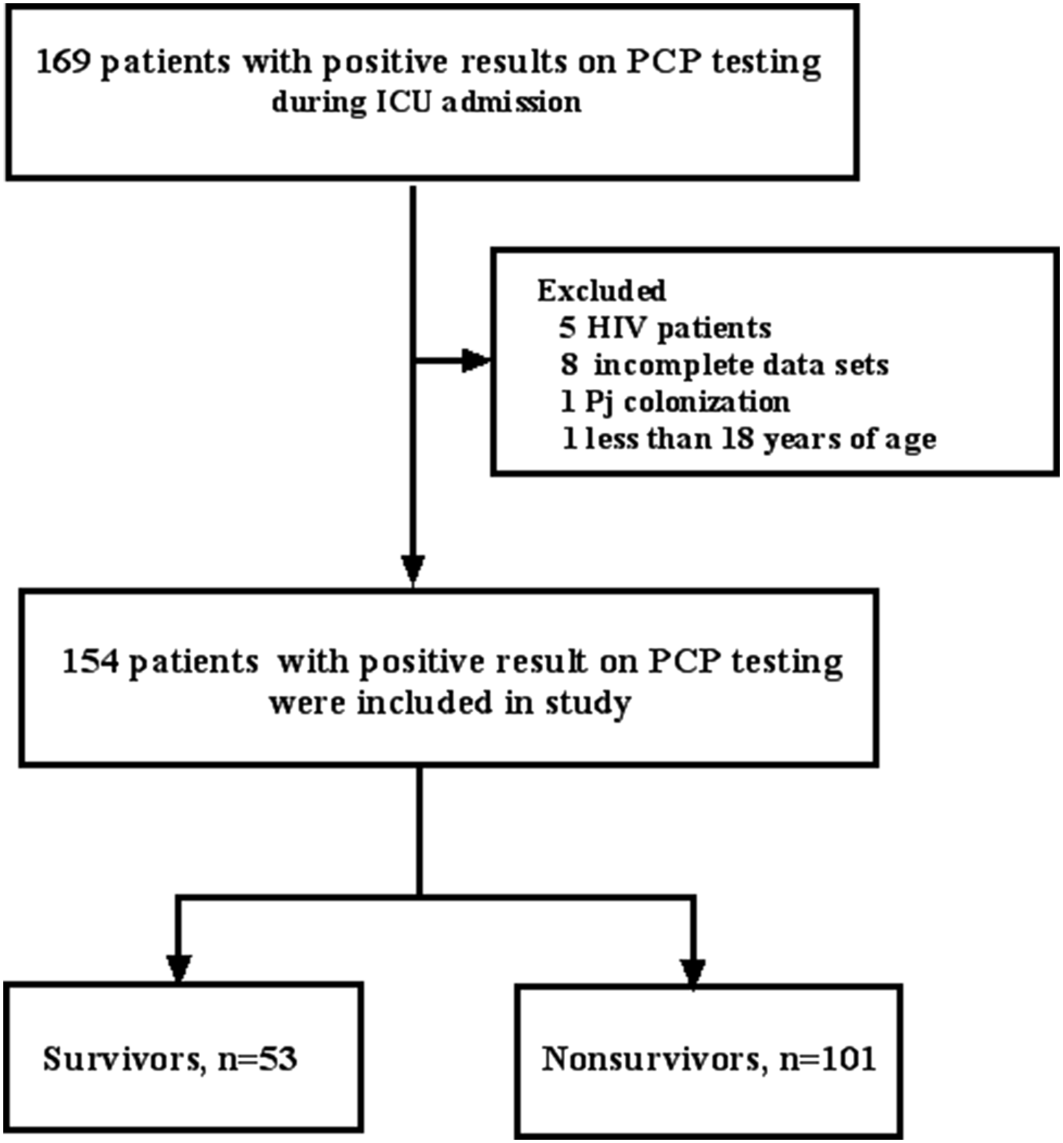
Study flow diagram. PCP, *Pneumocystis* pneumonia; TMP‐SMX, trimethoprim‐sulfamethoxazole

Between October 2012 and July 2020, 169 patients were confirmed positive for Pj by silver methoxamine staining or PCR. Finally, 154 patients were enrolled in the study (8 were excluded due to incomplete data, 5 were excluded due to HIV positivity, 1 was excluded due to Pj colonization, and 1 was younger than 18 years of age): 82 patients were treated at PUMCH, and 72 were treated at CJFH (Figure 1).

## 3 RESULTS

### 3.1 Demographics, baseline characteristics, treatments, and outcomes

The characteristics of the 154 HIV‐negative patients with PCP are shown in Table 1. The average age was 56 ± 15 years, and 50.6% of the study participants were male. Renal disease was the most common underlying disease (*n* = 36, 23.4%), followed by interstitial lung disease (ILD) (*n* = 25, 16.2%), dermatomyositis (*n* = 19, 12.3%), systemic lupus erythematosus (*n* = 15, 9.7%), vasculitis (*n* = 14, 9.1%), and hematologic malignancy (*n* = 11, 7.1%). In our study, 81.8% (*n* = 126) of patients were receiving glucocorticoids, and most of them also received combinations of immunosuppressive agents (68.2%, *n* = 105). PCP was diagnosed in 7, 122, and 25 patients by silver methoxamine staining, PCR, and a combination of the two methods, respectively. Samples positive for PCP on PCR included sputum (*n* = 7), tracheal aspirate (*n* = 21), and BALF (*n* = 119). Methenamine silver stain‐positive samples included sputum (*n* = 4), tracheal aspirate (*n* = 12), and BALF (*n* = 16). Pj co‐occurred with other lung infections. The majority (55.84%) were cytomegalovirus (CMV) infections, and there were 24 cases (15.58%) of pulmonary aspergillosis.

**TABLE 1:** Demographics, clinical characteristics, treatments, and complications of 154 HIV‐negative patients with *Pneumocystis* pneumonia

	Survivors, *N* = 53	Nonsurvivors, *N* = 101	*P* value
Age (years)	52 ± 16	58 ± 15	0.021
Gender, male	28 (52.8)	50 (49.5)	0.695
BMI, median (IQR)	22.3 (20.4–23.8)	21.83 (20.3–23.2)	0.319
APACHE II first day in ICU	17.4 ± 5.7	20.3 ± 5.7	0.003
Underlying disease			
Interstitial lung disease	9 (17.0)	16 (15.8)	0.855
Dermatomyositis	4 (7.5)	15 (14.9)	0.190
Systemic lupus erythematosus	7 (13.2)	8 (7.9)	0.293
Vasculitis	2 (3.8)	12 (11.9)	0.096
Renal disease	17 (32.7)	19(18.8)	0.055
Organ transplantation^a^	3 (5.7)	3 (3.0)	0.703
Solid tumor	2 (3.8)	6 (5.9)	0.847
Hematologic malignancy	3 (5.7)	8 (7.9)	0.851
Initial symptom			
Dyspnea	50 (94.3)	91 (90.1)	0.552
Fever	51 (96.2)	85 (84.2)	0.027
Cough	34 (64.2)	61 (60.4)	0.649
Laboratory test			
White blood cell counts, cells/μl	9180 ± 4431	8369 ± 3827	0.239
Lymphocyte counts, cells/μl, median (IQR)	570 (345–840)	430 (240–645)	0.014
G test, pg/ml, median (IQR)	266.0 (171.4–630.5)	278.0 (120.7–1061.5)	0.788
C‐reactive protein, mg/L, median (IQR)	14.5 (7.4–26.2)	15.6 (3.5–39.5)	0.736
Lactate dehydrogenase, U/L, median (IQR)	473.5 (353.3–795.5)	657.0 (524.0–886.0)	0.069
Albumin, g/dl, median (IQR)	29.8 (25.0–33.2)	30.0 (26.6–32.9)	0.650
Respiratory samples			
Sputum	1 (1.9)	6 (5.9)	0.459
Aspiration	7 (13.2)	17 (16.8)	0.556
BALF	45 (84.9)	78 (77.2)	0.259
Diagnostic methods			
PCR	50 (94.3)	97 (96.0)	0.941
Methenamine silver stain	7 (13.2)	25 (24.8)	0.093
Pulmonary coinfection			
CMV	31 (58.5)	55 (55.0)	0.679
Pulmonary aspergillosis	8 (15.1)	16 (15.8)	0.903
Treatment			
Duration from symptom onset to treatment, days, median (IQR)	9 (5–14)	7 (3–13)	0.111
Previous use of corticosteroid	48 (90.6)	87 (86.1)	0.427
Adjuvant steroid	44 (83.0)	82 (81.2)	0.780
High‐dose steroids (≥1 mg/kg/day prednisone equivalent)	21 (39.6)	57 (55.3)	0.047
Caspofungin	15 (28.3)	45 (44.6)	0.049
TMP‐SMX as initial regimen	53 (100.0)	100 (99.0)	1.000
Respiratory support			
PaO_2_/FiO_2_ (mmHg) on admission, median (IQR)	147.0 (116.5–197.0)	108.0 (82.5–163.5)	0.001
Mechanical ventilation			
IPPV	25 (47.2)	84 (83.2)	<0.001
NPPV	27 (50.9)	22 (21.8)	<0.001
Complications			
Shock	11 (20.8)	62 (61.4)	<0.001
Pneumothorax	4 (7.5)	11 (10.9)	0.506

*Note*: Values are expressed as *n* (%) or mean ± SD, unless stated otherwise.

Abbreviations: APACHE, Acute Physiology and Chronic Health Evaluation; BALF, bronchoalveolar lavage fluid; BMI, body mass index; CMV, cytomegalovirus; FiO_2_, inspiratory fraction of oxygen; ICU, intensive care unit; IPPV, intermittent positive pressure ventilation; IQR, interquartile range; NPPV, noninvasive positive pressure ventilation; PaO_2_, partial pressure of arterial oxygen; PCR, polymerase chain reaction; SD, standard deviation; TMP‐SMX, trimethoprim‐sulfamethoxazole.

^a^ Including hematopoietic stem cell transplantation and solid organ transplantation.

Only one patient did not initially receive trimethoprim‐sulfamethoxazole (TMP‐SMX). For the 60 patients who received caspofungin, the total treatment duration was 7 ± 6 days. Of the patients who received caspofungin, 13 patients received it as part of the initial combination treatment for PCP; 3 patients received it due to an established sulfa allergy; 2 patients received it due to a sulfa allergy discovered during the current hospitalization (1 patient had a blood cell count reduction, and 1 patient had a rash and vomiting); 1 patient received it due to gastrointestinal bleeding that prevented the patient from taking oral TMP‐SMX; 26 patients received it due to suspected invasive fungal infections; 6 patients received it combined with amphotericin B or voriconazole to treat aspergillosis; and 9 patients received it as a rescue treatment, though the clinical efficacy of caspofungin as a salvage therapy for PCP is still controversial.^21^

Of the 154 patients treated with PCP, 19 patients (12.3%) required continuous renal replacement therapy (CRRT), and 10 patients (6.5%) required extracorporeal membrane oxygenation (ECMO) support. The overall in‐hospital mortality rate was 65.6%. The mortality rate was 21.1% in patients requiring CRRT and 60.0% in patients requiring ECMO support. Nonsurvivors were more likely to receive invasive ventilation than survivors (83.2% vs. 47.2%, *P* < 0.001). The median duration of invasive ventilation was 11 days. Pneumothorax was detected in 15 patients, of whom 11 died.

### 3.2 Comparisons of clinical parameters of survivors and nonsurvivors

During hospitalization, 101 (65.6%) of the 154 patients died. As shown in Table 1, nonsurvivors with PCP were older (58 vs. 52 years, *P* = 0.021), were more likely to use high‐dose steroids (≥1 mg/kg/day prednisone equivalent, 39.62% vs. 55.34%, *P* = 0.047), use caspofungin during hospitalization (44.6% vs. 28.3%, *P* = 0.049), require invasive ventilation (83.2% vs. 47.2%, *P* < 0.001), develop shock during hospitalization (61.4% vs. 20.8%, *P* < 0.001), and develop pneumomediastinum (21.8% vs. 47.2%, *P* = 0.001) and had higher APACHE II scores at ICU admission (20.32 vs. 17.39, *P* = 0.003), lower lymphocyte counts (430 vs. 570 cells/μl, *P* = 0.014), and lower PaO2/FiO2 values (mmHg) on admission (108 vs. 147, *P* = 0.001).

There were no statistically significant differences in sex, body mass index (BMI), underlying diseases, previous corticosteroid use, white blood cell counts, respiratory samples, diagnostic methods, proportions of patients with concomitant CMV infections or pulmonary aspergillosis, durations from symptom onset to treatment, adjuvant steroid use, TMP‐SMX as the initial regimen, or pneumothorax between survivors and nonsurvivors.

### 3.3 Risk factors associated with in‐hospital mortality

To identify factors affecting PCP prognosis, clinical data were compared between survivors and nonsurvivors. Multivariate analysis of in‐hospital mortality was performed in non‐HIV‐infected patients with PCP. No meaningful interacting terms were found in this model, and there was no collinearity between any of the independent variables. The results of multivariate analysis of predictors of in‐hospital mortality are summarized in Table 2. Advanced age (odds ratio [OR] 1.03; 95% confidence interval [CI] 1.00–1.06; *P* = 0.024), a low oxygenation index on admission (OR 0.99; 95% CI 0.99–1.00; *P* = 0.014), and high‐dose steroids (≥1 mg/kg/day prednisone equivalent) (OR 2.29; 95% CI 1.07–4.90; *P* = 0.034) were associated with in‐hospital mortality. Meanwhile, we provided the data of sensitivity analysis using a *P* value cutoff of 0.1 for model selection in the supporting information, and the main study finding remains consistent after changing the *P* value cutoff.

**TABLE 2:** Univariate and multivariate analyses of independent factors associated with hospital mortality

Variable	Univariable model		Multivariable model	
	OR (95% CI)	*P* value	OR (95% CI)	*P* value
Age	1.03 (1.00–1.05)	0.024	1.03 (1.00–1.06)	0.024
Use of high‐dose steroids during hospitalization	1.97 (1.00–3.88)	0.049	2.29 (1.07–4.90)	0.034
Oxygenation index on admission	0.99 (0.99–1.00)	0.002	0.99 (0.99–1.00)	0.014
Lower lymphocyte counts	0.62 (0.34–1.14)	0.122	0.76 (0.39–1.47)	0.410

Abbreviations: CI, confidence interval; OR, odds ratio.

### 3.4 Subgroup analysis of patients who received caspofungin during hospitalization

A total of 60 patients received caspofungin, and 94 patients did not receive caspofungin. We analyzed the baseline data in the groups that did and did not receive caspofungin, and the results are shown in Table 3. There were no significant differences in the baseline data between these two groups. As shown in Table 4, patients who received caspofungin were more likely to require invasive ventilation (80.0% vs. 64.9%, *P* = 0.044), were more likely to develop shock in the hospital (56.7% vs. 41.5%, *P* = 0.066), and had a higher in‐hospital mortality rate (75.0% vs. 59.6%, *P* = 0.049).

**TABLE 3:** Demographics of 154 patients with and without caspofungin

	With caspofungin (*n* = 60)	Without caspofungin (*n* = 94)	*P* value
Age (years)	54.0 ± 16.9	57.0 ± 14.3	0.266
Gender, male	27 (45.0)	51 (54.3)	0.263
BMI, median (IQR)	21.2 (20.3–23.1)	22.5 (20.4–23.4)	0.195
APACHE II first day in ICU	19.7 ± 6.0	19.1 ± 5.7	0.564
Lymphocyte counts, cells/μl, median (IQR)	430 (275–648)	465 (270–800)	0.288
CMV coinfection	33 (55.0)	53 (56.4)	0.956
Pulmonary aspergillosis coinfection	7 (11.7)	17 (18.1)	0.284
Previous use of corticosteroid	52 (86.7)	83 (88.3)	0.764
Adjuvant steroid	50 (83.3)	76 (80.9)	0.697
PaO_2_/FiO_2_ (mmHg) on admission, median (IQR)	123 (83.0–175.0)	130 (94.8–180.3)	0.231
Invasive ventilation on admission	16 (26.7)	38 (40.4)	0.081

*Note*: Values are expressed as *n* (%) or mean ± SD, unless stated otherwise.

Abbreviations: APACHE, Acute Physiology and Chronic Health Evaluation; BMI, body mass index; CMV, cytomegalovirus; FiO_2_, fraction of inspiratory oxygen; IQR, interquartile range; PaO_2_, arterial partial pressure of oxygen; SD, standard deviation.

**TABLE 4:** Clinical outcome of 154 patients with and without caspofungin

	With caspofungin (*n* = 60)	Without caspofungin (*n* = 94)	*P* value
Invasive ventilation during hospitalization	48 (80.0)	61 (64.9)	0.044
Shock during hospitalization	34 (56.7)	39 (41.5)	0.066
Died during hospitalization	45 (75.0)	56 (59.6)	0.049

*Note*: Values are expressed as *n* (%).

## 4 DISCUSSION

In this bicentric, retrospective, case–control study, we assessed predictive factors of in‐hospital mortality in non‐HIV‐infected patients with PCP admitted to the ICU and found that advanced age, high‐dose steroids (≥1 mg/kg/day prednisone equivalent), and a low oxygenation index on admission were predictors of in‐hospital mortality. Use of caspofungin during hospitalization might contribute to the poor prognosis in the ICU.

The diagnosis and treatment level of autoimmune diseases in our hospital is in the forefront of the country, so a large number of patients with autoimmune diseases who take steroids and immunosuppressants seek medical treatment due to PCP infection. Therefore, the proportions of autoimmune diseases and steroid exposure were higher than other reports. In contrast, there are relatively fewer cancer, organ transplant, or hematopoietic stem cell transplantation patients, so our results may be more applicable to patients with autoimmune diseases.

The mortality rate of patients in this study was 65.6%, which was consistent with the mortality rate of 33.3%–69.3% reported in previous studies.^8^, ^9^, ^10^, ^11^, ^12^, ^13^, ^14^ CMV coinfection is very common (*n* = 86, 55.8%), which suggests that a CMV diagnostic test should be performed for every patient diagnosed with PCP.^22^ Delayed treatment was previously considered a poor prognostic factor for patients with PCP.^23^ However, the present study did not detect a difference in the timing of treatment initiation between survivors and nonsurvivors. To a certain extent, this is because of the presence of selection bias. Our hospital has an excellent reputation with regard to the treatment of respiratory diseases, and therefore, many financially able patients come to our hospital for treatment. We believe that empirical treatment is very important to decrease the mortality rate in non‐HIV‐infected patients with PCP.^24^ In our study, only three patients received PCP prophylaxis. Although prophylaxis for PCP is recommended for HIV patients, the efficacy of prophylaxis for non‐HIV patients with PCP has not been well established,^25^, ^26^ especially for the patients using corticosteroids and immunosuppressive agents.

We found that older age was related to a high in‐hospital mortality rate among non‐HIV‐infected patients with PCP. Age is widely used for risk stratification, and older age is associated with a worse PCP prognosis in both HIV‐positive and HIV‐negative subjects.^17^, ^27^, ^28^, ^29^, ^30^, ^31^, ^32^

A low oxygenation index on admission was identified as a predictor of in‐hospital mortality. This reflects the disease severity, including a poor general condition and the need for intensive care, which makes patients more susceptible to nosocomial infections and other complications. In addition, invasive ventilation usually indicates that the patient has severe hypoxemia, which can cause multiple organ failure, such as acute respiratory distress syndrome.^33^ We performed endotracheal intubation for patients with a low oxygenation index. According to reports, the mortality rate for non‐HIV PCP can reach 62%–76% if a ventilator is needed,^34^, ^35^ and our mortality rate was 77%, which might be explained by ventilator‐associated pneumonia (VAP). To correct hypoxia, ventilator parameters are generally increased, which often leads to VAP.^36^ In our study, the proportion of patients who developed pneumomediastinum was 16.9% (*n* = 26), and 84.6% (*n* = 22) of the patients who developed pneumomediastinum died. Of the 22 patients who died, only 1 did not receive invasive ventilation. Therefore, barotrauma, including pneumothorax, pneumomediastinum, and pneumohypoderma caused by invasive ventilation, might also explain the poor prognosis.^37^, ^38^, ^39^

In this study, use of high‐dose steroids (≥1 mg/kg/day prednisone equivalent) was identified as a risk predictor of in‐hospital mortality. Several studies have demonstrated that adjunctive corticosteroid therapy is beneficial for patients who have HIV and symptoms of moderate to severe PCP. However, there have been no interventional trials in HIV‐negative patients with PCP, and the results of several retrospective studies are conflicting. Two previous studies^25^, ^40^ analyzed the effect of corticosteroids on non‐HIV patients with severe PCP, and the results indicated that adjunctive corticosteroid therapy failed to reduce the in‐hospital mortality. Similar to our study, Lemiale et al performed a pooled analysis of 139 non‐HIV ICU patients with severe PCP and found that high‐dose steroid treatment (≥1 mg/kg/day prednisone equivalent) was an independent predictor of ICU mortality.^41^ However, in a retrospective study, Pareja et al reported that a daily prednisone dose of 60 mg or more resulted in a better outcome than lower doses of prednisone.^42^ Therefore, the decision to add corticosteroids for a non‐HIV patient with PCP must be individualized, particularly if hypoxemia is present.^43^ However, large prospective studies are needed to draw accurate conclusions.

Our subgroup analysis results showed that the use of caspofungin is associated with increased in‐hospital mortality and invasive ventilation rates, which is similar to the findings of a previous study.^44^ There are currently no large‐scale prospective studies on the treatment of PCP with caspofungin. It remains controversial whether the use of caspofungin improves patient outcomes. One previous study found that HIV‐negative patients with PCP did not respond to caspofungin treatment.^45^ However, since 2006, the use of caspofungin for the treatment of PCP has been reported in Chinese and international studies.^46^, ^47^ Some hospitals already use caspofungin as a second‐line treatment for PCP. Although there was no significant difference in the baseline data of patients who did and did not receive caspofungin in our study (Table 3), it should be noted that some patients who used caspofungin did so due to initial treatment failure or combined fungal infections. The baseline condition of these patients may have been worse, which may explain why our conclusion is different from those drawn in the abovementioned studies. At present, there are no large studies that have demonstrated the efficacy of caspofungin in the treatment of PCP. Given our sample size, we only present the results for discussion. Whether the use of caspofungin can improve PCP patient prognosis needs to be determined in large‐scale prospective trials.

### 4.1 Limitations

Our study had some limitations. First, this was a retrospective study conducted in two centers without follow up. Therefore, it is not possible to draw definite conclusions. Second, the two hospitals in this study are renowned for their rheumatology and clinical immunology departments, which may have led to a high proportion of patients with autoimmune diseases in the study population; therefore, the results may not be truly reflective of the entire population at high risk for PCP in China. The numbers of patients in the subgroups stratified by underlying disease were small; therefore, differences in prognoses among patients undergoing different types of immunosuppression could not be detected. Thus, prospective studies with larger sample sizes are necessary. Third, the respiratory specimens consisted of sputum, tracheal aspirate, and BALF. Therefore, the heterogeneity of these sources may have affected the final analysis.

## 5 CONCLUSION

In this study, the vast majority of non‐HIV‐infected patients with PCP had inflammatory diseases and were treated with glucocorticoids. The mortality rate was high, and predictive factors of a poor prognosis were advanced age, high‐dose steroids (≥1 mg/kg/day prednisone equivalent), and a low oxygenation index on admission. The use of caspofungin during hospitalization might have no contribution to the prognosis of non‐HIV infected patients with PCP in ICU.

## CONFLICT OF INTEREST

The authors declare that they have no competing interests.

## ETHICS STATEMENT

To maintain patient anonymity and confidentiality, we did not collect identifying information, and only the researchers involved in this study evaluated the data. The need to obtain written informed consent was waived by the ethics committee due to the retrospective nature of the analysis.

## AUTHOR CONTRIBUTIONS

QZ and LW had full access to all of the data in the study and take responsibility for the integrity of the data and the accuracy of the data analysis. YW, XH, and TS contributed substantially to the study design, data analysis and interpretation, and the writing of the manuscript.

## Supporting information


**Data S1.** Supplementary Appendix

## Data Availability

The datasets used and/or analyzed during the current study are available from the corresponding author upon reasonable request.
