# Supplementary material for: Non‐HIV‐infected patients with Pneumocystis pneumonia in the intensive care unit: A bicentric, retrospective study focused on predictive factors of in‐hospital mortality
Source: Clin Respir J. 2022 Jan 10;16(2):152–61. doi: 10.1111/crj.13463 (PMC9060091; doi:10.1111/crj.13463)
Supplement: Supplementary file 1 — Data S1. Supplementary Appendix [file CRJ-16-152-s001.docx]

**Supplementary Appendix**

This appendix has been provided by the authors to give readers additional information about their work.

Supplement to: Qingyuan Zhan, Li Weng, Yuqiong Wang, et al. Non-HIV-infected patients with pneumocystis pneumonia in the intensive care unit: a bicentric, retrospective study focused on predictive factors of in-hospital mortality. The Clinical Respiratory Journal. DOI: 10.1111/CRJ.13463

**Supplementary materials**

Statistical method description page 1
